# Complete Genome Sequence of *Delftia acidovorans* RAY209, a Plant Growth-Promoting Rhizobacterium for Canola and Soybean

**DOI:** 10.1128/genomeA.01224-17

**Published:** 2017-11-02

**Authors:** Benjamin J. Perry, Jordyn Bergsveinson, Dinah D. Tambalo, Christopher K. Yost, Nurul H. Khan, Mike Whiting

**Affiliations:** a Department of Biology, University of Regina, Regina, Saskatchewan, Canada; b Lallemand Specialties, Inc., Saskatoon, Saskatchewan, Canada

## Abstract

Herein, we report the genome sequence of *Delftia acidovorans* strain RAY209, a plant growth-promoting rhizobacterium that is used in commercial inoculants for canola and soybean. The genome of RAY209 has a consensus of 6,528,879 bp and an estimated 5,721 coding sequences.

## GENOME ANNOUNCEMENT

*Delftia acidovorans* strain RAY209 was isolated from a canola rhizosphere from western Canada and is used commercially as an inoculant for canola and soybean. This strain promotes root development in canola and improved crop yield based on several field trials (1, 2). RAY209 is also included with *Bradyrhizobium japonicum* as a commercial mixed-inoculant for soybean plants to promote seed emergence, increased nodule number (3), and yield.

Genomic DNA was isolated using an UltraClean Microbial DNA isolation kit (MoBio), and DNA sequencing was performed by Genome Quebec (Montreal, Canada) using PacBio single-molecule real-time (SMRT) sequencing technology (4). Contigs were assembled by Genome Quebec using the hierarchical genome-assembly process (HGAP) workflow (5), resulting in genome coverage of 129×. Annotation was performed through the NCBI Prokaryotic Genome Annotation Pipeline (6). Average nucleotide identity (ANI) analysis using Jspecies (7) was used to compare the RAY209 genome to other sequenced genomes of *Delftia*.

The genome has a consensus length of 6,528,879 bp, a G+C content of 66.6%, 5,721 coding sequences, 15 rRNAs, and 80 tRNAs. Strain RAY209 shares very high ANI values (99.9%) with *D. acidovorans* 2167 (GenBank accession number JOUB01000005), *D. acidovorans* SPH-1 (accession number CP000884), *D. acidovorans* CCUG 15835 (accession number AGYY00000000), *D. acidovorans* CCUG 274B (accession number AGYX01000012), and *D. acidovorans* CM13 (accession number CP017420).

The genome of RAY209 contains genes for motility and chemotaxis. Further analysis using antiSMASH (8) identified gene loci for resorcinol, terpenes, and a bacteriocin, which may have antimicrobial properties (9, 10). Comparison of functional capacity of the above listed *D. acidovorans* genomes in Rapid Annotations using Subsystems Technology (RAST) (11) reveals that RAY209 has unique genes related to cell wall and capsule synthesis, osmotic stress, iron acquisition and metabolism, and niacin-choline transport and metabolism. Adaptation of the cell wall and synthesis of osmoregulated periplasmic glucans are expected to assist the cells under unfavorable osmotic conditions or desiccation, providing support for the suitability of RAY209 as a seed inoculant. The role and uptake of iron by rhizobacterium has been well investigated, suggesting that root bacteria can influence the iron-uptake machinery of their host plant (12). Niacin and choline are growth factors exuded by plants; thus, the capacity of RAY209 to uptake and metabolize provides a competitive advantage for their growth and activity. Further, genes related to this functional subsystem have been identified among the family of genes common to both plants and prokaryotes (13). Further exploration of the genetic and functional capacity of RAY209 will be critical to increase understanding of its plant growth-promoting activity.

### Accession number(s).

The genome of *D. acidovorans* RAY209 was deposited in the GenBank/EMBL database with the accession number CP022656.
